# Quantification of forward scattering based on the analysis of double‐pass images in the frequency domain

**DOI:** 10.1111/aos.14122

**Published:** 2019-05-03

**Authors:** Joan A. Martínez‐Roda, Carlos E. García‐Guerra, Fernando Diaz‐Doutón, Jaume Pujol, Antoni Salvador, Meritxell Vilaseca

**Affiliations:** ^1^ Centre for Sensors, Instruments and Systems Development (CD6) Universitat Politècnica de Catalunya (UPC) Terrassa Spain; ^2^ Ophthalmology Service Hospital Universitari Mútua de Terrassa Terrassa Spain

**Keywords:** aberrations, cataracts, double‐pass, forward intraocular scattering

## Abstract

**Purpose:**

To assess forward intraocular scattering by means of a new parameter (Frequency Scatter Index, FSI_3_) based on the analysing double‐pass retinal images in the frequency domain, which minimizes the impact of aberrations on the results.

**Methods:**

A prospective observational study was carried out in the Department of Ophthalmology, Hospital Universitari Mútua de Terrassa (Spain) on a group of 19 patients diagnosed with nuclear cataracts of various LOCSIII grades and a control group (CG) with nine healthy eyes. We recorded double‐pass retinal images with a custom set‐up based on a high‐sensitivity digital camera. The FSI_3_ was then computed using spatial frequencies below three cycles per degree. A preliminary validation of the FSI_3_ was performed on an artificial eye and two eyes of volunteers with and without commercial diffusers, and under defocus.

**Results:**

The FSI_3_ was hardly affected by defocus values up to 2.50 D. The mean (and standard deviation) FSI_3_ values were as follows: for the CG, 1.19 (0.21); and for LOCSIII grades nuclear opacity 1, 2 and 3, 1.30 (0.12), 1.62 (0.21) and 1.85 (0.21), respectively. Eyes with cataracts showed FSI_3_ values significantly different than healthy eyes (p = 0.001). A good correlation (*ρ* = 0.861, p < 0.001) was found between the FSI_3_ and objective scatter index provided by a commercial instrument.

**Conclusion:**

Since aberrations have little impact on the FSI_3_, the light scatter assessment becomes less dependent on the patient's refractive error compensation and the presence of higher‐order aberrations. The FSI_3_ can further the clinical and scientific understanding of forward intraocular scattering.

## Introduction

Forward intraocular scattering is a phenomenon of human vision that can currently be quantified by psychophysical and objective methods (Westheimer & Liang 1994; Franssen et al. 2006). When commercial devices have been used to compare both types of methods in patients with posterior capsule opacification (Hirnschall et al. 2014) and in patients with cataracts (Martínez‐Roda et al. 2016a, b), significantly divergent results of forward light scatter have been obtained. Double‐pass (DP) systems and the objective scatter index (OSI) (Artal et al. 2011) have been widely used to classify cataracts in clinical environments (Vilaseca et al. 2012). An increase in the OSI has been observed in eyes with keratoconus (Leonard et al. 2016), and significant correlations have been found between the OSI and the logMAR in patients after corneal transplantation (Kamiya et al. 2015). The OSI compares the integrated intensity contained in a ring between 0.20° and 0.33° (degrees), that is, between 12 and 20 min of arc, with the integrated intensity contained in the central area of the DP image within 1 min of arc. However, the presence of aberrations can affect the OSI (Miao et al. 2014). Ginis et al. (2012) recently presented an experimental system to reconstruct a wide‐angle version of the point spread function (PSF) of the eye, where the reconstructed curve contains data up to 8° and may be used to quantify intraocular scattering. Due to its characteristics, the system is restricted to an area of 2 mm in exit pupil diameter and does not provide information on aberrations.

In this study, we propose a new parameter to quantify intraocular scattering based on the analysis of the information contained in the DP image at eccentricities between 0.59° and 2.35°, in order to minimize the impact of aberrations on the results. The region selected is hardly affected by aberrations while containing the effects of intraocular scattering (van den Berg et al. 2009). Furthermore, the DP image used incorporates the overall effects of scattering within an exit pupil diameter of 4 mm. The parameter is computed in the frequency domain applied to the lowest part of the spectrum of the DP image, specifically the range comprised between 0 and 3 cycles per degree (cpd) of the ocular modulation transfer function (MTF).

## Methods

To analyse the performance of the parameter proposed in a clinical setting, we conducted a study in patients diagnosed with nuclear cataracts of different severity, and in a control group (CG) of subjects with healthy eyes. This prospective study was approved by the *Hospital Universitari Mútua de Terrassa* ethics committee and conformed to the tenets of the Declaration of Helsinki (Tokyo revision, 2004). The study included patients who were scheduled for cataract check‐ups and a CG of volunteers from our university. All patients signed a consent form after the purpose of the study had been explained. Inclusion criteria were the diagnosis of nuclear cataracts, with refractive spherical equivalent from −6.00 to +6.00 dioptres (D) and astigmatism below 3.00 D. Exclusion criteria were the diagnosis of any other eye condition, presence of corneal opacities and history of ocular trauma or surgery.

Manifest refraction and corrected distance visual acuity (CDVA) were assessed under physiological pupil conditions. After pupil dilation with 0.2 ml of 1% tropicamide, a slit lamp examination was performed; the ophthalmologist determined the type and grade of cataract in terms of lens opacity [nuclear opacity 1, 2 and 3 (NO1, NO2 and NO3)] within the LOCS III classification system.

The protocol included measurement of forward intraocular scattering with the HD Analyzer instrument (Visiometrics S.L., Cerdanyola del Vallès, Barcelona, Spain) by means of the OSI (Artal et al. 2011) and with the DP experimental instrument described in section Experimental setup and image acquisition by means of the new Frequency Scatter Index (FSI_3_).

### Experimental set‐up and image acquisition

**Figure 1:**
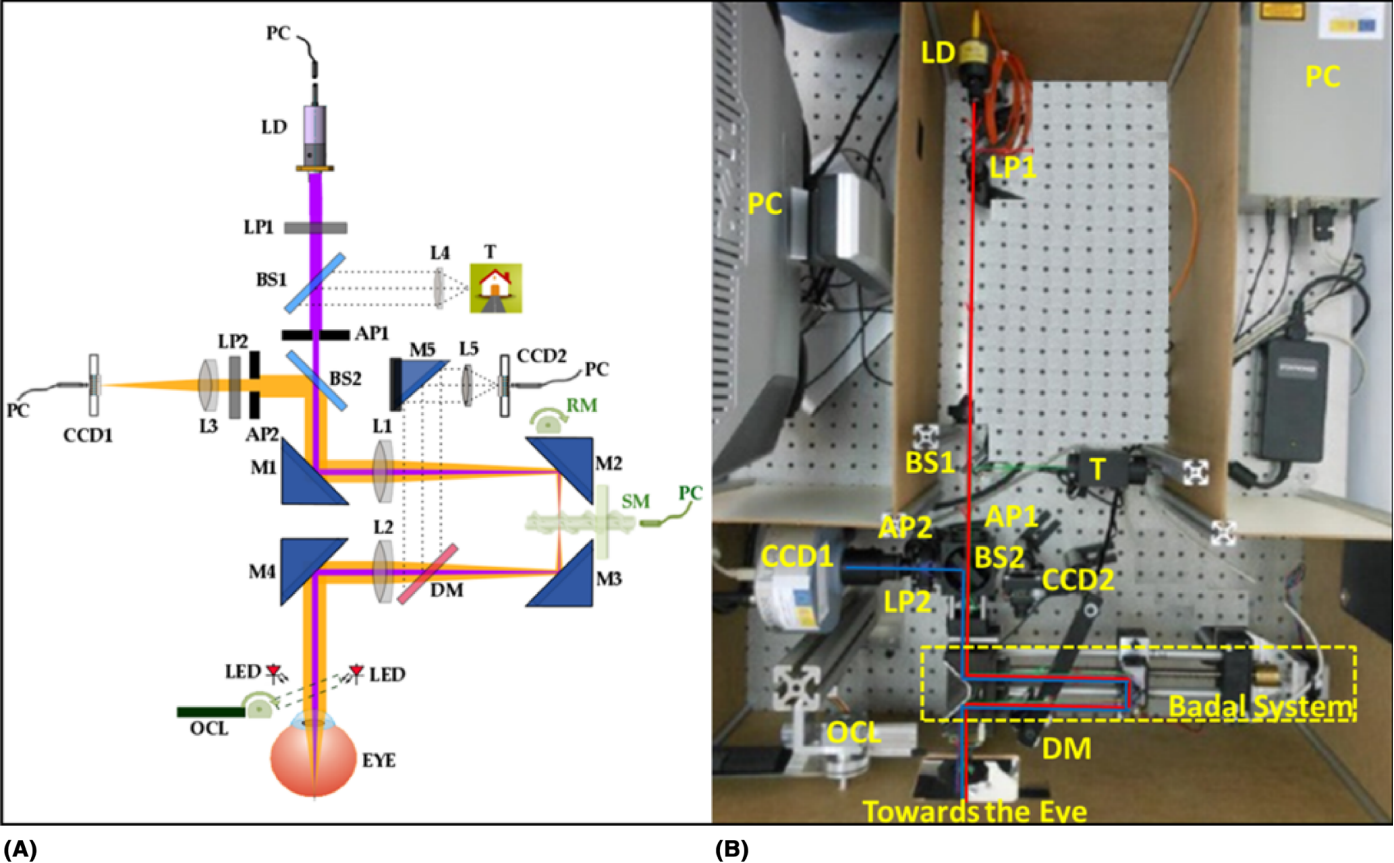
Layout (A) and picture (B) of the double‐pass set‐up. AP = pupil, BS = beam splitters, CCD = Charge‐coupled device, DM = dichroic mirror, L = lens, LD = laser diode, LP = linear polarizer, M = mirror, OCL = occluder, PC = personal computer, RM = rotating motor, SM = step motor, T = test. In purple, the optical path of the first‐pass, and in orange, the optical path of the second‐pass.

We used an experimental DP instrument to acquire DP images as described elsewhere (Artal et al. 2011). In this study, the diameter of the exit pupil was set to 4 mm for the whole procedure. Briefly, the instrument records the retinal image corresponding to a point‐source object in the near‐infrared, consisting of a laser diode (MC7800C‐M‐004S‐7A10, wavelength 780 nm. Monocrom S.L., Vilanova i la Geltru, Spain) coupled to an optical fibre, after diffuse reflection on the ocular fundus and double pass through the ocular media. The experimental device used is shown in Fig. 1.

During measurements, collimated light from the laser source (LD) was transmitted by the linear polarizer LP1 before reaching the circular diaphragm AP1 of 2 mm in diameter that acted as entrance pupil. After being transmitted by the beam splitter BS2 and reflected by mirror M1, the light interacted with the Badal system formed by two identical lenses (L1 and L2) of 150 mm in focal length. This configuration was used to correct the spherical refractive error of patients between −6.00 and +6.00 D, modifying the optical distance between the lenses with the remote‐controlled step motor that moved forward or backward mirrors M2 and M3. After lens L2, the light was reflected by mirror M4 before reaching the pupil plane of the eye and focusing the light onto the retina of the patient.

After interacting with the ocular fundus, the reflected light followed an optical path identical to the first pass until it reached the beam splitter BS2, which reflected the light coming from the eye towards the exit pupil of 4 mm in diameter formed by diaphragm AP2. The light was then filtered by the linear polarizer LP2, which removed corneal reflections in combination with the crossed polarizer LP1 located within the first‐pass optical path. Finally, lens L3 of 50 mm in focal length focused the DP spot on the sensor of the imaging device CCD1 (Electron Multiplying CCD Luca^EM^ R, Andor Technology^TM^, Belfast, UK). This 14‐bit‐depth cooled camera recorded DP images with a pixel resolution of 8 μm × 8 μm (0.55 min of arc) using a sensor of 8 mm × 8 mm, providing single photon detection sensitivity at all eccentricities. This method ensures the measurement of all light reflected back from the retina, which contains the effects of forward light scatter, light diffusion in the choroid and aberrations.

Additionally, a rotating motor was mounted on mirror M2. The vibration provided by the motor allowed M2 to act as a scanning mirror to obtain images with reduced speckle (Hofer et al. 2001; Sanabria et al. 2014). During measurements, the pupil position in the image provided by camera CCD2 (UI‐1226LE‐M; IDS Imaging Development Systems GmbH, Obersulm, Germany) monitored the alignment between the optical axis of the system and the eye. The imaging device processed the light at 900 nm from the light emitting diodes placed in front of the eye after imaging the pupil plane with lenses L2 and L5, mirrors M4 and M5, and dichroic mirror. The LD, motor SM, and cameras CCD1 and CCD2 were controlled remotely using the personal computer through a customized program in java (Oracle, Java SE and NetBeans, version 8.0.2; Redwood City, CA, USA, 2015) developed in the open‐source platform Micro‐Manager (Edelstein et al. 2010). Once acquired, the DP images were processed offline using the open‐source software imagej (Schneider et al. 2012).

The light reaching the eye was measured with a radiometer IL 1700 (International Light Technologies, Peabody, MA, USA). The irradiance for the 780 nm LD measured at the corneal plane was of 0.48 W/m^2^, a value considerably lower than the maximum permissible exposure of 14.45 W/m^2^ defined in the corresponding standards (International Electrotechnical Commission. Safety of laser products – part 1: Equipment classification and requirements. IEC 60825‐1:2014, Edition 3, May 2014).

The Badal system of the instrument automatically corrected the patients’ spherical refraction, while astigmatism was corrected with an external cylindrical lens. A set of six consecutive DP images, each obtained with an exposure time of 200 ms, was recorded, and the average of the images was calculated. Another image was acquired with the eye removed from the system and subtracted from the first one to obtain the image used for the analysis.

### Intraocular scattering assessment

#### Preliminary assumptions

Double‐pass (DP) images contain the overall effects of both aberrations and scattering (Diaz‐Santana & Dainty 2001; Díaz‐Doutón et al. 2006). However, it seems reasonable to consider that the information about aberrations and scattering is spatially distributed: while the effects of aberrations are preponderant in the area closest to the peak of maximum intensity, the ones of scattering predominate in more eccentric areas.

**Figure 2:**
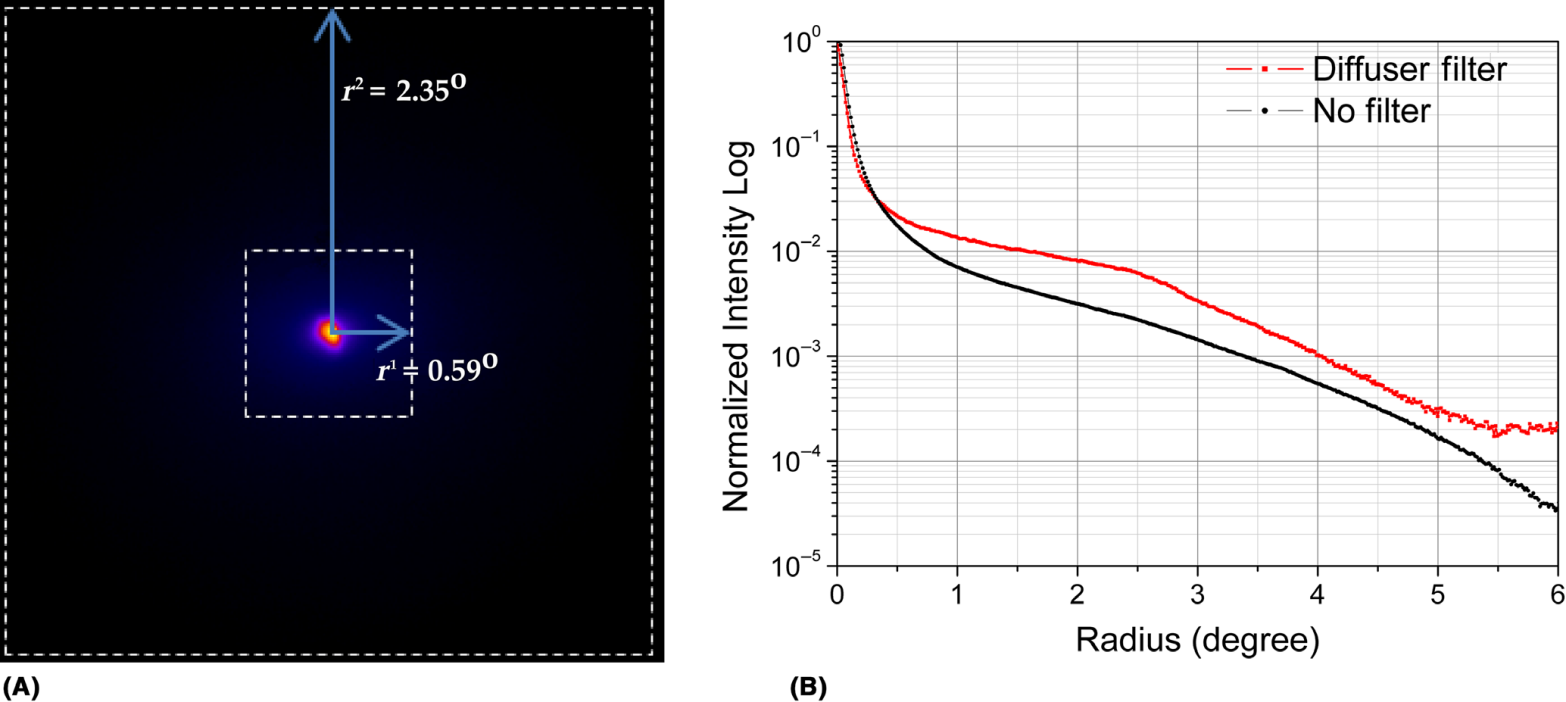
Pseudocolour double‐pass (DP) image (A): marked by dashed white lines with regions of interest of 128 × 128 pixels (r1 = 0.59°) and 512 × 512 pixels (r2 = 2.35°). Radial average profile in logarithmic scale of the DP image (B): corresponding to the image on the left (black) and with a diffuser filter in front of the same eye (red); dashed lines indicate the position of r1 and r2.

To implement the FSI_3_, two MTF curves are obtained after applying a Fourier transform to two cropped versions of the DP image with squared regions of interest of extensions r1 and r2, with r1 < r2, and a common central position defined by the peak with maximum intensity of the image (Fig. 2). In the region defined by r1, the effects of aberrations mask the ones of scattering. On the contrary, the effects of aberrations are negligible compared to the ones of scattering in the borders of the region defined by r2. In this work, we analyse MTF curves obtained from regions with r1 = 0.59° (128 × 128 pixels) and r2 = 2.35° (512 × 512 pixels) of the DP image to obtain the FSI_3_.

**Figure 3:**
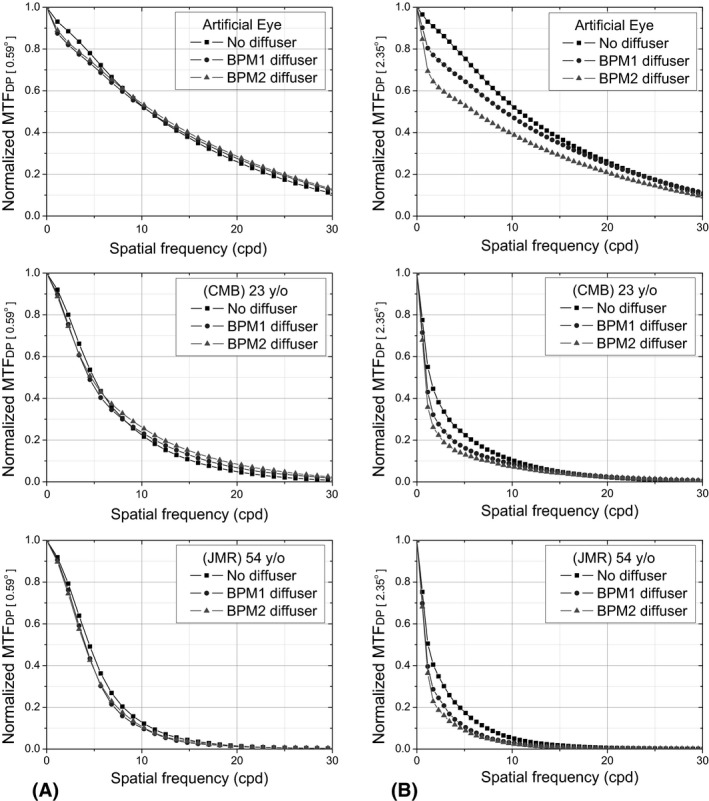
Normalized mean radial profile of the MTF_DP_ without diffuser (squares) and with diffuser BPM1 (circles) and BPM2 (triangles), regions of interest with sizes r1 = 0.59° (A), and r2 = 2.35° (B), for the artificial eye (top) and the two eyes of volunteers aged 23 (middle) and 54 (bottom). BPM = Black Pro‐Mist.

To evaluate the effects of intraocular scattering in the two proposed regions of interest (ROI), we used the commercially available diffusers Black Pro‐Mist (BPM) 1 and 2 (Tiffen, Hauppauge, NY, USA), widely employed to simulate incipient and moderate cataracts, respectively (De Wit et al. 2006). Each diffuser was placed in front of an artificial eye consisting of a lens of 50 mm in focal length and a cardboard working as a retina, as well as in front of two human eyes from two volunteers aged 23 and 54 years. The graphs on the left of Fig. 3 show the normalized radial average of the MTF curves obtained with the ROI of size r1 (MTF_DP[0.59⁰]_). As can be seen, the diffuser filters barely affect the MTFs obtained. The graphs on the right show the MTF curves obtained with the ROI of size r2 (MTF_DP[2.34⁰]_), where, by contrast, the effect of the diffuser filters is noticeable.

Some authors have presented models where the effects of aberrations and scattering are included in the MTF of the eye in a multiplicative form (Rodríguez et al. 2007). Assuming this as true, and considering that MTF_DP[0.59⁰]_ is basically affected by aberrations and that MTF_DP[2.35⁰]_ is affected by both aberrations and diffusion, we can use the ratio between the MTFs obtained from the two ROIs (MTF_*F*[r2–r1]_) to estimate the effect of diffusion in the ocular media as follows:

$$\frac{{\text{MTF}}_{\mathrm{DP}[r2]}\left(\nu \right)}{{\text{MTF}}_{\mathrm{DP}[r1]}\left(\nu \right)}\propto ∼{\text{MTF}}_{F[r2-r1]}\left(\nu \right).$$

**Figure 4:**
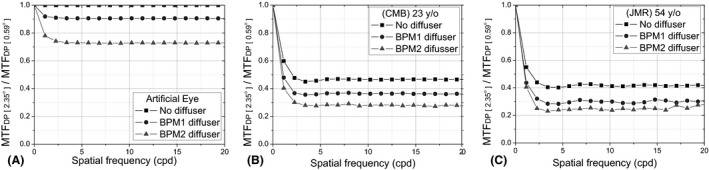
MTF_F[r2– r1]_ (*ʋ*) without diffuser (squares) and with diffuser BPM1 (circles) and BPM2 (triangles) for the artificial eye (A) and the two eyes of volunteers aged 23 (B) and 54 (C) years. BPM = Black Pro‐Mist.

The ratios MTF_F[r2‐r1]_ for the artificial and the two human eyes under the presence of the different diffusers considering ROIs with sizes r1 = 0.59° and r2 = 2.35° are shown in Fig. 4. As observed, in all three cases a similar pattern is observed, that is, an abrupt drop at lower frequencies and a stable behaviour beyond approximately 3 cpd. The former is related to the amount of induced intraocular scattering, and as expected, it was higher for the older eye (Rozema et al. 2010; Martínez‐Roda et al. 2016a).

#### Definition of the Frequency Scatter Index (FSI_3_)

The aforementioned results verify the assumption that information on intraocular scattering is contained mainly at lower spatial frequencies. Accordingly, a novel parameter to quantify intraocular scattering (FSI_3_) is proposed as follows:

$${\text{FSI}}_{3}=\frac{n}{\sum_{0}^{n(\nu <3\mathrm{cpd})}{\text{MTF}}_{F[r2-r1]}\left(\nu \right)}-1$$

where subindex 3 indicates that only frequency values between 0 and 3 cpd of the MTF_DP_ are included. Specifically, in our set‐up *n* was set to 6, since these discrete numbers of frequencies were available in the interval considered – from 0 to 3 cpd. In accordance with the formula, FSI_3_ values over 1 are obtained in the presence of intraocular scattering. In order to establish a parameter with a null value in the absence of intraocular scattering, 1 is finally subtracted from the equation. Moreover, the parameter depends on the size of the selected ROIs. In our case, the weight of the information contained is evaluated up to an angle of 2.35° compared to that of 0.59°.

#### Impact of intraocular scatter on FSI_3_

**Figure 5:**
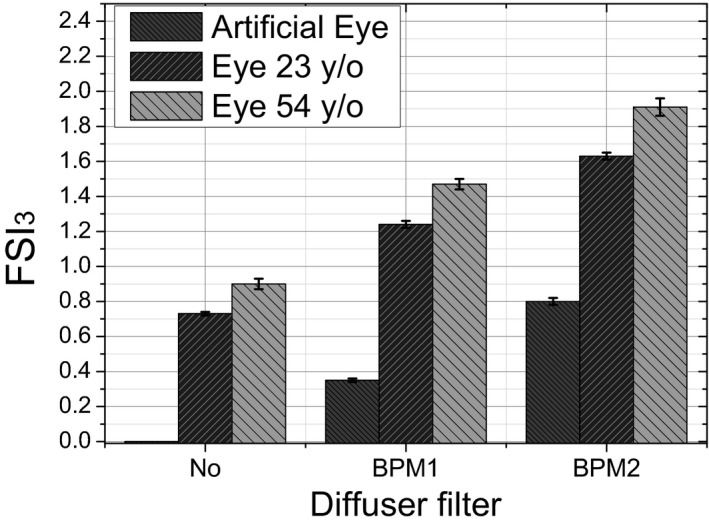
Bar chart plot with the mean and the standard deviation values of five repeated measurements of the Frequency Scatter Index parameter for the artificial and the two eyes of volunteers aged 23 and 54 years without diffuser (No) and with diffusers Black Pro‐Mist 1 and 2.

To corroborate its correct performance, the proposed parameter was used to assess intraocular scattering in the artificial and human eyes of volunteers aged 23 and 54 years with and without diffusers BPM1 and BPM2. Five repeated measurements were obtained for each eye. Figure 5 shows the mean and the standard deviation of this computation. The FSI_3_ increases for all eyes in correlation with the level of scattering. As expected, the older eye shows higher FSI_3_ values, whereas the lowest values correspond to the artificial eye.

The fact that the parameter does not get a 0 value for the 23‐year‐old subject without scattering may be attributable to lateral diffusion in the layers of the retina or even in the choroid due to the wavelength used (Delori & Pflibsen 1989; Williams et al. 1994). Even so, the differences between the two individuals are small compared to the increase with diffuser filters.

#### Impact of defocus on FSI_3_

**Figure 6:**
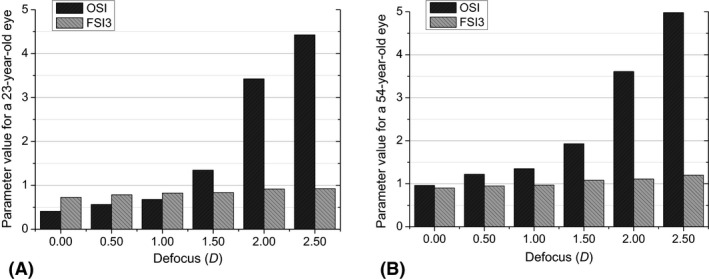
Frequency Scatter Index (measured with the experimental set‐up) and objective scatter index (measured with the commercial HDA instrument) at the best focus position and with induced defocus up to 2.50 D in 0.50 D steps: (A) volunteer aged 23 and (B) volunteer aged 54.

The performance of the FSI_3_ under defocus was also studied as a preliminary validation. To this end, measurements from the two subjects aged 23 and 54 years were again obtained under different amounts of induced defocus, specifically between 0.00 and 2.50 D in steps of 0.50 D induced by means of the Badal system available in the experimental set‐up. The difference between values of FSI_3_ without defocus and values with a maximum defocus of 2.50 D were of 0.20 and 0.30 for the two subjects, respectively. Figure 6 shows the FSI_3_ values for 0.00 D and other defocus conditions, proving that FSI_3_ values increased minimally with defocus, which demonstrates the robustness of this parameter in the presence of this low‐order aberration. In contrast, the OSI increased significantly from 1.00 D defocus, probably because the defocus causes a sharp decrease in the PSF peak. In this case, the maximum differences were 4.02 (in terms of OSI) at a 2.50 D defocus for both subjects.

The observed behaviour was already expected because the FSI_3_ includes the region of the DP image between 0.59° and 2.35°, much larger than the OSI region (from 0.20° to 0.33°). The OSI region is very close to the central peak of the image and thus more affected by aberrations, whereas scattering dominates the behaviour of the region used to calculate the FSI_3_.

### Statistical analysis

The Shapiro–Wilk test was used to evaluate the normal distribution of the FSI_3_, OSI and CDVA. An independent sample t‐test was used to compare the mean between the CG and the eyes with cataracts and between each LOCSIII subgroup (NO1, NO2 and NO3). Since the OSI data did not meet the criteria for normal distribution, the Mann–Whitney *U*‐test (*z*) was used to compare the data between subgroups NO1, NO2 and NO3, and the Spearman rank correlation coefficient (*ρ*) was calculated to assess the relationships between the FSI_3_, the OSI and the CDVA. Chi‐square tests were used to compare the proportion of sex and age within the groups. Descriptive data are shown as the mean ± standard deviation (SD) for normally distributed variables and median and interquartile range for non‐normally distributed variables. A value of p < 0.05 was considered statistically significant.

## Results

Patient demographics are shown in Table 1. Nine control eyes of nine healthy observers and 19 eyes of 19 patients diagnosed exclusively with aged‐related nuclear cataracts were finally included in the study. No statistically significant differences were noted between the CG and cataract group or among the LOCSIII‐grade subgroups (NO1, NO2 and NO3) regarding sex, right/left eye or manifest subjective refraction. There was a significant difference in terms of averaged age between the CG and the cataract group (p < 0.001); no age difference existed among the LOCSIII subgroups (p > 0.05).

**Table 1:** Demographic of the control and cataract groups according to sex, eye, age and manifest subjective refraction (spherical equivalent)

Parameter	CG	Cataract group		
		Subgroups of LOCSIII grade		
		NO1	NO2	NO3
Sex (*n*)				
Male	5	4	6	2
Female	4	2	2	3
Eye (*n*)				
Right	4	4	3	2
Left	5	2	5	3
Age (years)				
Mean (SD)	55 (3)	71 (8)	71 (6)	73 (6)
Range (min max)	50 58	56 80	60 77	66 80
Spherical equivalent (D)				
Mean (SD)	−0.38 (1.61)	+0.75 (1.30)	−0.77 (2.85)	+1.10 (2.59)
Range (min max)	−3.50 +1.50	−1.38 +2.63	−5.13 +2.37	−2.50 +4.50

CG = control group, NO = nuclear opacity, SD = standard deviation.

The FSI_3_ was significantly higher (p = 0.001, *t*‐test) in the cataract group than in the CG. Table 2 shows how the averaged FSI_3_ increases with the LOCSIII grade. We found statistically significant differences between subgroups NO1 and NO2 (p = 0.005), but not between NO2 and NO3 (p = 0.050). The OSI was also significantly higher in the cataract group compared to the CG (p < 0.001, Mann–Whitney *U*‐test), and significant differences were found between subgroups NO1 and NO2 (p = 0.002) and NO2 and NO3 (p = 0.003). The CDVA was also significantly higher in the cataract group than in the CG (p < 0.001), but the differences between NO1 and NO2 and between NO2 and NO3 were not statistically significant (p > 0.05).

**Table 2:** FSI_3_, OSI and CDVA values of the control and cataract groups

Parameter	CG	Cataract group		
		Subgroups of LOCSIII grade		
		NO1	NO2	NO3
FSI_3_				
Mean (SD)	1.18 (0.20)	1.30 (0.12)	1.62 (0.21)	1.85 (0.21)
OSI				
Median (IQR)	1.30 (0.24)	1.63 (0.47)	3.26 (0.78)	4.06 (1.83)
CDVA (LogMAR)				
Median (IQR)	0.00 (0.02)	0.26 (0.57)	0.35 (0.19)	0.40 (0.29)

CDVA = corrected distance visual acuity, CG = control group, FSI_3_ = Frequency Scatter Index, IQR = interquartile range, NO = nuclear opacity, OSI = objective scatter index, SD = standard deviation.

**Figure 7:**
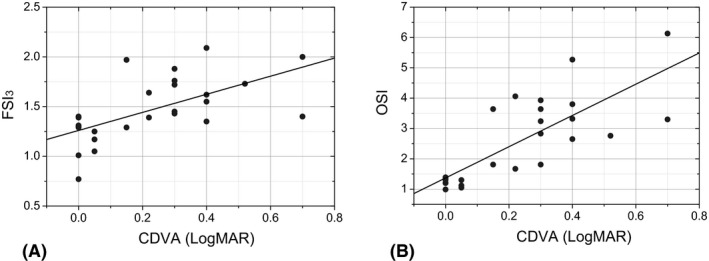
Scatterplot with the individual values of corrected distance visual acuity (*x*‐axis) and Frequency Scatter Index (A) (*r* ^2^ = 0.258) and objective scatter index (B) (*r* ^2 ^= 0.310) (*y*‐axis).

In addition, we found very strong correlations between the FSI_3_ and the OSI (*ρ* = 0.861, p < 0.001) and between the OSI and the CDVA (*ρ* = 0.672, p < 0.001), but moderate between FSI_3_ and CDVA (*ρ* = 0.508, p = 0.006; Fig. 7). A similar correlation was found between the FSI_3_ and the OSI when the CG (*ρ* = 0.733, p = 0.025) and the cataract group (*ρ* = 0.773, p < 0.001) were separately analysed.

**Figure 8:**
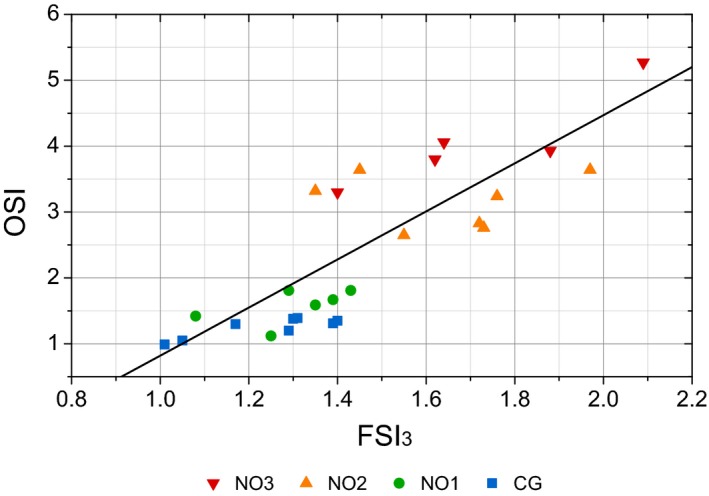
Scatterplot with the individual values of Frequency Scatter Index (*x*‐axis) and objective scatter index (*y*‐axis) (*r* ^2^ = 0.698). Markers in colours by subgroups of LOCSIII grade (NO1, NO2 and NO3) and control group (CG).

Finally, the scatterplot for the individual FSI_3_ and OSI values with different scales for each parameter is shown in Fig. 8. Interestingly, FSI_3_ and OSI share approximately 70% of information about intraocular scattering.

## Discussion

The analysis of intensity distribution in the DP image has been widely used to obtain information about the contribution of aberrations and intraocular scattering on the retinal image. The proposed FSI_3_ quantifies scattering from a wide region of the DP image which subtends an angle of 2.35° and applies only to low spatial frequencies, that is, below 3 cpd. The results show that the new parameter is very sensitive to the presence of scattering caused by commercial diffusers. Furthermore, preliminary validations demonstrate that only the measurement for the artificial eye provides a null FSI_3_ value, whereas human eyes with cataracts show a scattering value comparable to that induced with the BPM1 diffuser.

When computing the FSI_3_ parameter, the information where the effects of aberrations are dominant is filtered to minimize its impact. In consequence, the defocus values up to 2.50 D in the DP image have little impact on the FSI_3_ when measuring scattering, particularly in comparison with the widely used OSI. Specifically, in the case of the OSI, an induced defocus of the DP image above 1.00 D results in a considerable overestimation of scattering, according to this and previous studies (Artal et al. 2011).

With regard to the results of patients with cataracts, forward and backward light scatter measurements cannot be used interchangeably. Slit lamp grading is based on backward light scatter, whereas the FSI_3_ measures forward scattering, although a strong correlation exists between the FSI_3_ and NO. The better correlation between NO and OSI is probably due to OSI scattering overestimates caused by the presence of higher‐order aberrations in eyes with mature cataracts (Rocha et al. 2007; Lee et al. 2008). On the other hand, FSI_3_ and OSI share approximately 70% of scatter in our sample of patients with cataracts (Fig. 8). At this point, it should be borne in mind that in this study an experimental device has been used, whose configuration and components slightly differ from commercially available DP instruments. Especially the 14‐bit‐depth cooled camera allows obtaining information with very low noise level. The suitability of devices with different configurations or characteristics to quantify forward scattering with the new parameter has not been subject of this study. Another limitation of this study is the limited number of patients. Therefore, it would be also necessary to expand it to determine the FSI_3_ values in healthy patients and for the different degrees of severity of cataracts.

In conclusion, this study presents a novel parameter for measuring intraocular scattering computed from DP images in the frequency domain with a minimal impact of aberrations. The main objective of the FSI_3_ would be to obtain measurements less dependent on patients’ refractive error compensation and higher‐order aberrations. The parameter is sensitive to scattering induced by diffusers and caused by cataracts. Furthermore, it discriminates the information on scatter and aberrations contained in the DP image more effectively than the OSI. The parameter FSI_3_ evaluates the information contained in an area that subtends from 0.59° to 2.35° of the DP image which includes the intraocular scattering and the scatter that occurs in the inner layers of the retina. The use of a different wavelength with less penetration would allow diminishing the influence of this scatter. Even so, the results indicate that this has a much lower value than that caused by intraocular diffusion in patients with cataract. In addition, the variability between healthy individuals is also small compared to the presence of cataracts, although it should be studied in a greater number of individuals.

Additionally, the integration of these two objective parameters results in a greater understanding of these visual phenomena with regard to different ocular conditions in clinical and research settings (Jinabhai et al. 2012; Kamiya et al. 2015; Leonard et al. 2016). Further studies will analyse a larger number of patients with different patterns of scatter, especially posterior subcapsular and cortical cataracts, as well as in different corneal pathologies that cause scattering and age‐matched controls. Furthermore, the suitability of the proposed methodology using eight‐bit‐depth cameras, which are nowadays included in commercially available DP instruments, will be also studied. Regardless the fact that we used six discrete frequencies for the computation of the FSI_3_ as this was the number of values available within the frequency range considered in our system, the methodology is still valid for any other frequency sampling between 0 and 3 cpd available in any other set‐up.
